# Novel Biallelic Variant in the *BRAT1* Gene Caused Nonprogressive Cerebellar Ataxia Syndrome

**DOI:** 10.3389/fgene.2022.821587

**Published:** 2022-03-10

**Authors:** Yiming Qi, Xueqi Ji, Hongke Ding, Ling Liu, Yan Zhang, Aihua Yin

**Affiliations:** ^1^ Prenatal Diagnosis Center, Guangdong Women and Children Hospital, Guangzhou, China; ^2^ Maternal and Children Metabolic-Genetic Key Laboratory, Guangdong Women and Children Hospital, Guangzhou, China; ^3^ Clinical Medicine College, Guangzhou Medical University, Guangzhou, China

**Keywords:** *BRAT1*, nonprogressive cerebellar ataxia syndrome, synonymous variant, intron retention, prenatal diagnosis

## Abstract

Recessive mutations in *BRAT1* cause lethal neonatal rigidity and multifocal seizure syndrome (RMFSL), a phenotype characterized by neonatal microcephaly, hypertonia, and refractory epilepsy with premature death. Recently, attenuated disease variants have been described, suggesting that a wider clinical spectrum of *BRAT1*-associated neurodegeneration exists than was previously thought. Here, we reported a 10-year-old girl with severe intellectual disability, rigidity, ataxia or dyspraxia, and cerebellar atrophy on brain MRI; two *BRAT1* variants in the *trans* configuration [c.1014A > C (p.Pro338 = ); c.706delC (p.Leu236Cysfs*5)] were detected using whole-exome sequencing. RNA-seq confirmed significantly decreased *BRAT1* transcript levels in the presence of the variant; further, it revealed an intron retention between exon 7 and exon 8 caused by the synonymous base substitute. Subsequent prenatal diagnosis for these two variants guided the parents to reproduce. We expand the phenotypic spectrum of *BRAT1*-associated disorders by first reporting the pathogenic synonymous variant of the *BRAT1* gene, resulting in clinical severity that is mild compared to the severe phenotype seen in RMFSL. Making an accurate diagnosis and prognostic evaluation of *BRAT1*-associated neurodegeneration is important for reproductive consultation and disease management.

## Introduction

Variations in *BRAT1* (BRCA1-associated ataxia telangiectasia mutated activator 1) are initially recognized as the cause of lethal neonatal rigidity and multifocal seizure syndrome (RMFSL; OMIM#614498), which is characterized by neonatal microcephaly, intractable seizures, hypertonia, and early demise. Subsequently, neurodevelopmental disorder with cerebellar atrophy and with or without seizures (NEDCAS; OMIM#618056) caused by biallelic *BRAT1* variants were reported and redefined the description of “lethality.” Recently, a milder clinical form that manifests as nonprogressive cerebellar ataxia (NPCA) was described in some childhood-onset patients (Mahjoub et al., 2019), suggesting that a wider phenotypic spectrum of *BRAT1*-associated neurodegeneration exists than was previously thought.

Physiological functions of the disease-causing gene *BRAT1* are diverse (Fernandez-Jaen and Ouchi, 2016). It encodes a protein that interacts with the tumor suppressor gene BRCA1 at its C-terminus and binds to ATM1, considering a master controller of the cell cycle signaling pathways required for cellular responses to DNA damage (Ouchi and Ouchi, 2010). *BRAT1* can form a complex with an ATPase domain-containing protein, BRP1 (*BRAT1* Partner 1), and prevent transcriptional silencing at methylated genomic regions (Zhang et al., 2016). It may also be involved in cell growth and apoptosis (Straussberg et al., 2015). *BRAT1* deficiency secondary to biallelic *BRAT1* mutations may increase the glucose metabolism, reduce the mitochondrial reactive oxygen species (ROS) concentration, deteriorate cell growth and migration, and induce neuronal atrophy (Puffenberger et al., 2012; Saunders et al., 2012; So and Ouchi, 2014; Wolf et al., 2015). The complexity and extensiveness of the *BRAT1* gene function are the biological basis for the huge phenotypic heterogeneity. However, the exact mechanism by which variations in *BRAT1* trigger neurodegeneration and to what extent a defect in ATM function contributes to this disease are unknown.

In this study, we first reported the clinical course of a proband with NPCA caused by novel compound heterozygous *BRAT1* variants, which include a negligible pathogenic synonymous variant. Functional studies confirmed the effect of them on transcription. The result provided a theoretical basis and guidance for this family in reproductive genetic counseling and prenatal diagnosis. Furthermore, we summarized all published cases with *BRAT1* variation, which provide insight into the clinical–genetic correlation and the pathophysiology of the disease.

### Clinical Report

A 10-year-old girl who presented severe intellectual disability and poor motor ability was transferred to our genetics center for consultation due to the family’s reproductive plan.

**FIGURE 1:**
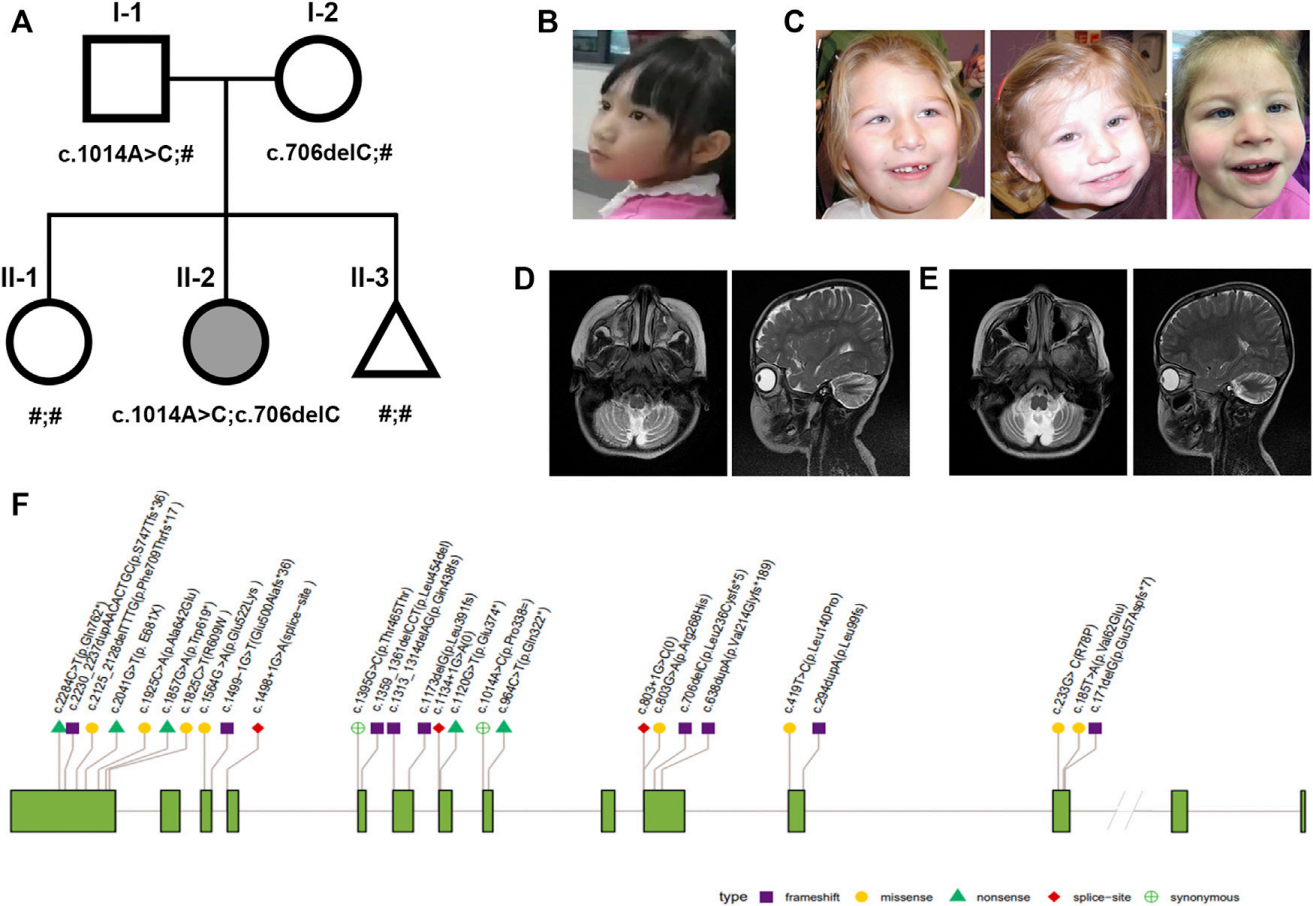
Nonprogressive cerebellar ataxia syndrome phenotype and pedigrees. **(A)** Pedigrees of the family with NPCA profiled in this study. The proband is marked in black. **(B–C)** Facial appearance of the proband is not specific (left); typical facial dysmorphisms in reported BRAT1-associated NPCA including epicanthal folds, a high arched palate, etc [right, Ref (Srivastava et al., 2016)]. **(D–E)** Brain MRI of the proband at the age of 4 showed typical cerebellar atrophy (left), which almost showed no progress at 9 years (right). **(F)** Distribution of reported pathogenic variants in *BRAT1*. Annotations include the following: exon (grass–green square); variation types: frameshift (purple square), missense (yellow circle), nonsense (green triangle), red diamond (splice site), and synonymous (green cross circle).

The patient was the second child of nonconsanguineous parents of Chinese and had a healthy adolescent sister (pedigree in Figure 1A). There were unremarkable findings during her prenatal, perinatal, and neonatal courses. Physical examination at birth revealed a normal height, normal weight, and normal head 101 circumference (51 cm; 48th percentile).

However, global developmental delay was presented initially a few months later. She developed head control at 6 months and could not sit until 15 months. At 2.5 years, she could only babble, make consonant sounds, and communicate her needs by crying and gazing. After an individualized neurorehabilitation therapy, she was able to sit briefly and pull to stand at 3 years. One year later, she could stand independently for a few minutes and walk with a walker. At 5 years, she could say 5–10 word phrases that were dysarthric, identify people who were in constant contact with her, and follow simple commands. Meanwhile, behavior with episodes of impulsivity and irritability began to appear since then. She attended special schooling for rehabilitation training but with poor performance until last re-evaluation and was considered to have severe intellectual disability with a mental age of less than 3 years.

Until now, she could stand alone and walk slowly with limited support in a rigid- and broad-based gait with flexed arm posture (Supplementary Videos S1,2). She also presented slight dysmetria, which interferes in fine motor skills during the performance of tasks (Supplementary Video S3). Although her facial expression shows relative paucity (Figure 1B), no evident *BRAT1*-assoicated dysmorphisms, such as epicanthal folds, high arched palate, fifth finger clinodactyly, or single palmar crease, were observed (Figure 1C). Her cranial nerves were intact except pendular nystagmus. Motor exam has shown mild hypertonia and resistance during extension, but tendon reflexes were normal.

Cranial magnetic resonance imaging (MRI) spectroscopy was first performed at the age of 4, which recorded cerebellar atrophy (Figure 1D). In the following years, she showed stable cerebellar atrophy on serial neuro-MRI (Figure 1E). Electroencephalography was uniformly negative.

Laboratory examination included serum tests for alpha-fetoprotein (AFP), inborn errors of the metabolism, amino acids, very long-chain fatty acids, acylcarnitine and carnitine profiles, infectious work-up, TORCH (toxoplasmosis, rubella, cytomegalovirus, and herpes simplex virus) tests, urine organic acid analyses, and conventional indicators in cerebrospinal fluid (CSF) analysis, which were all within normal limits. We also ruled out the possibility of serine biosynthesis defects and nonketotic hyperglycinemia by normal serine and glycine. After precluding aneuploidy and copy number variations (CNVs), we conducted a Trio WES on the family.

## Materials and Methods

### Genetic Investigations

Genomic DNA was extracted using a Qiagen DNA blood mini kit (Qiagen GmbH, Hilden, Germany). Library preparation and target enrichment were performed using a SureSelectXT Clinical Research Exome kit (Agilent Technologies, Santa Clara, CA) according to the manufacturer’s specifications. Then, Trio WES was performed using 2 × 150 bp in the paired end mode of the NextSeq 500 platform (Illumina, San Diego, CA) to obtain an average coverage of above 110x, with 97.6% of target bases covered at least 10x. Sequence quality analysis and filtering of mapped target sequences were performed with the ‘varbank’ exome and genome analysis pipeline v.2.1 as described previously (Vetro et al., 2020). Analysis of genetic results was based on the genomic variation database (http://dgv.tcag.ca/dgv/app/home), DECIPHER database (https://decipher.sanger.ac.uk/), and OMIM database (http://www.ncbi.nlm.nih.gov/omim). The found variants were further verified by Sanger sequencing in fetuses and parents.

### RNA Sequence

Total RNA was extracted from peripheral blood samples using Qiagen blood RNA extraction kit I (QIAGEN, United States); the procedures and standards were performed according to the manual. After complete property control of RNA and quality control of RNA concentration and purity are qualified, 1 μg of RNA is aspirated for mRNA library construction. The mRNA library was constructed using a TIANSeq Fast RNA Library Kit (Illumina, United States) according to the manufacturer’s instructions, where mRNA was purified and enriched from 1 μg of the total RNA samples and then fragmented about 250 bp, and the index adapter was added. Finally, using a high-fidelity enzyme amplifies the library. After quality control, the libraries were sequenced on an Illumina HiSeq 4,000 platform.

### Chorionic Villus Sampling

Fetal samples were collected by chorionic villus sampling at 13 weeks of gestation. The procedure was performed using the transabdominal approach. Under aseptic conditions, an 18 or 20 gauge spinal needle was inserted into the placenta under continuous ultrasound guidance. A 20 cc syringe containing the collection media is attached to the end of the needle once the stylet is removed. Negative pressure is created, and the needle is moved up and down through the placenta, collecting the tissue (Jones and Montero, 2021).

## Results

### Genetic Findings

**FIGURE 2:**
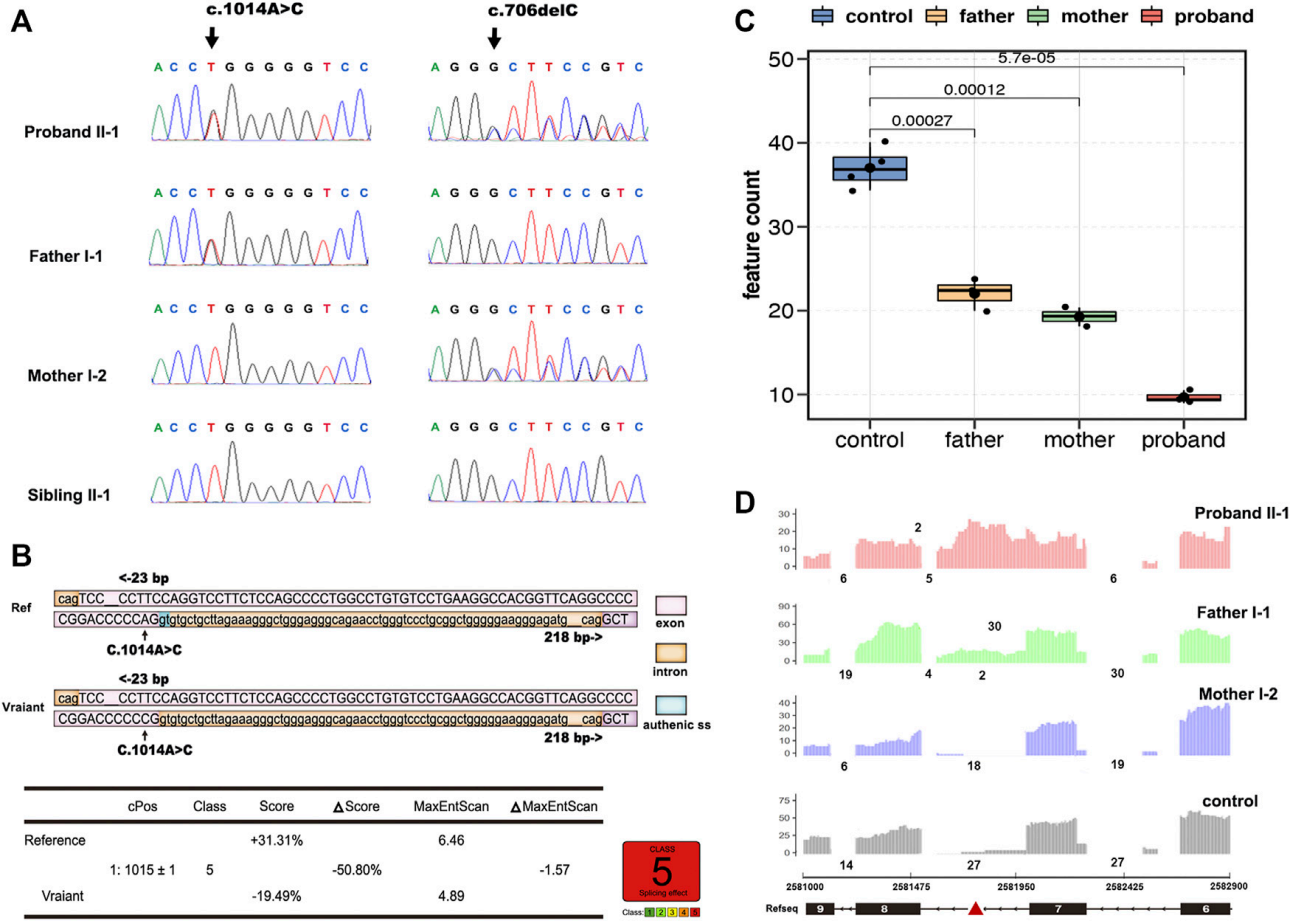
Overview of genetic testing results of BRAT1. Sanger sequencing of BRAT1 variants. **(A)** Chromatograms demonstrated the compound heterozygous status of c.1014A > C and c.706delC in the BRAT1 gene in the proband, the proband’s father, and the proband’s mother. The position of the variant or the corresponding wild-type nucleotide is labeled with black arrows. **(B)** Software predictions strongly suggest that the substitution of c.1014A > C might cause aberrant splicing. **(C)** Groups were significantly different from each other (controls vs. proband, *p* < 5.7*10^−5^; controls vs. father, *p* < 2.7*10^−4^; controls vs. mother, *p* < 1.2*10^−4^; and two-tailed unpaired *t*-test). **(D)** RNA-seq read coverage shows aberrant retention of intron 7 of BRAT1 in the proband and in the heterozygous (c.1014A > C) father relative to wild-type controls.

Whole-exome sequencing analysis identified compound heterozygous mutations in the *BRAT1* gene, c.706delC (p.Leu236Cysfs*5), and a synonymous variant, c.1014A > C (p.Pro338 = ), which were confirmed by sanger sequencing and segregated with the disorder in her family (Figure 2A).

Neither both the variants were reported in public databases nor their functional impact was examined. The deletion variant c.706delC (p.Leu236Cysfs*5) in exon 5 was predicted to lead to a frameshift and to cause loss of the full-length protein (821 amino acids) due to truncation after the first 236 residues. It is predicted to be pathogenic (VarSome, https://varsome.com/ and ClinVar https://www.ncbi.nlm.nih.gov/clinvar/).

The other variant, c.1014A > C (p.Pro338 = ) in exon 7/8, is not conserved among species, and the amino acid pro at the position 338 of *BRAT1* protein is not changed as well; however, varSEAK analysis predicts that the mutation may cause the classic splicing site c.1015 + 1 to be skipped (Figure 2B). Thus, RNA-seq was exerted to identify potential splicing defects associated with the variants in the NPCA case.

*BRAT1* expression levels in RNA-seq showed a trend toward lower expression in heterozygous parents and the compound heterozygous proband and confirmed significantly decreased *BRAT1* transcript levels in the presence of the variant (Figure 2C), consistent with NMD of the mutant transcript. Meanwhile, *BRAT1* expression was higher in the synonymous variants’ father than in the frameshift variants’ mother, although the difference was not statistically significant (Figure 2C). Furthermore, RNA-seq analyses identified significantly increased retention of intron 7 of *BRAT1* in the proband and the heterozygous father relative to the heterozygous mother and wild-type control samples (Figure 2D). It was presumed to be the effect of NM_152743.3 c.1014A > C (p.Pro338 = ) mutation. Altogether, these results suggest that both the variants participate in the pathogenesis of NPCA.

### Prenatal Diagnosis for Reproduction and Follow-Up

The mother of the proband underwent prenatal diagnosis at 12 weeks of gestation during her third pregnancy using chorionic villus sampling. Fortunately, Sanger sequencing showed that the fetus inherited neither of the above sites (Figure 1A). Prenatal and neonatal courses of the fetus were uncomplicated. A male neonate 2,560 g in weight and 51 cm high was delivered at the term, without microcephaly (HC 348 mm). In the following years, his head circumference has grown consistently within the 95th centile. Other growth parameters, strength, reflexes, and sensation were normal until the last visit at 18 months.

## Discussion

A total of 30 patients with clinical manifestations ranging from RMFSL to NPCA had been identified as homozygous or compound heterozygous variants in *BRAT1* (Table 1, Figure 1F). In *BRAT1*-related disorders, cerebellar hypoplasia seemed not congenital (Wolf et al., 2015; Srivastava et al., 2016; Celik et al., 2017). Clinical classifications depend on the rate of progression of atrophy after birth determined. Nearly 75% of them present with severe RMFSL, with almost all accompanied by multifocal or refractory epilepsy or even intrauterine jerks (Celik et al., 2017). Epilepsy occurs in 41.6% of NEDCAS/NPCA patients, mostly before 3 years of age. It had been reported that a few NPCA cases, which mapped to the SCA15 locus on chromosome 3pter, might develop spasticity or focal dystonia with increasing age (Dudding et al., 2004). However, in NPCA caused by BRAT1, including the case here, later-onset spasticity is extremely rare on long-term follow-up.

**TABLE 1:** Clinical features of individuals with previously described BRAT1 variants besides the present study.

NO.	Gender	Variant	Effect	Pedigree	Reported condition	Associated clinical phenotypes	Electroencephalogram	Brain MRI	Ref
1	M	c.185T > A	p.Val62Glu	Sibling wih NO.2	24 years’ old, graduated from college	Mild intellectual disability, ataxia, motor development delay, language delay, gaze-evoked nystagmus	Not offered	9 years: stable isolated cerebellar atrophy	Mahjoub et al. (2019)
		c.185T > A						16 years: stable isolated cerebellar atrophy	
2	M	c.185T > A	p.Val62Glu	Sibling wih NO.1	7 years’ old	Mild intellectual disability, ataxic, motor development delay, dysarthria, gaze-evoked nystagmus	Not offered	3 years: stable isolated cerebellar atrophy	Mahjoub et al. (2019)
		c.185T > A						5 years: stable isolated cerebellar atrophy	
3	F	c.638dupA	p.Val214Glyfs^a^189	Sibling wih NO.4	10 years’ old	Hypotonia, microcephaly, dysmetria and truncal titubation, ataxic, intellectual disability, ataxia, and cerebellar atrophy, head circumference, global developmental delay, bilatera l5th finger clinodactyly	Not offered	Prominent cerebellar interfolial spaces, which remained unchanged from 2, 3 years	Srivastava et al. (2016)
		c.803+1G > C	Splice site						
4	F	c.638dupA	p.Val214Glyfs^a^189	Sibling wih NO.3	6 years’ old	Hypotonic, dysmetria, microcephaly, dysarthric speech and pendular nystagmus, global developmental delay, activity-induced tremor, bilateral 5th finger clinodactyly	Normal	Progressive enlargement of the cerebellar interfolial spaces, cerebellar atrophy	Srivastava et al. (2016)
		c.803+1G > C	Splice site						
5	F	c.294dupA	p.Leu99Thrfs^a^92	Sporadic	6 years’ old	Seizures, hypertonia, microcephalic, generalized axial and peripheral hypertonia and hyper-reflexia, motor development delay, language delay	Mainly left-sided temporo-occipital epileptiform discharges and absence of a posterior dominant rhythm	3 mo: decreased myelination and thin corpus callosum	Mundy et al. (2016)
		c.1925C > A	p.Ala642Glu					3 years: right temporal lobe encephalomalacia and cerebellar and vermis hypoplasia	
6	M	c.1564G > A	p.Glu522Lys p.Val214Glyfs^a^189	Sporadic	4.5 years’ old	Microcephaly, hypertonia, progressive encephalopathy never presented seizures	Normal	19 and 48 mo: moderate progressive cerebellar atrophy	Fernández-Jaén et al. (2016)
		c.638dupA							
7	F	c.638dupA	p.Val214Glyfs^a^189	Sporadic	4 years 4 mo old	Right esotropia, mild optic nerve hypoplasia, with decreased visual acuity bilaterally, moderate appendicular rigidity, dyspraxia, global developmental delay, bilateral5th finger clinodactyly	Showed frequent 3–4 Hz generalized spike and wave complexes (without clinical correlate)	5 mo: normal 21 mo and 4 years 3 mo: enlargement of the cerebellar interfolial spaces compatible with cerebellar atrophy and mildly delayed myelination	Srivastava et al. (2016)
		c.419T > C	p.Leu140Pro						
8	F	c.1857G > A	p.Trp619^a^	Sibling wih NO.15	4 years and 4 mo	Drug-resistan seizures, microcephaly, developmental delay	Multifocal epileptiform activity	Not offered	Smith et al. (2016)
		c.2125_2128delTTTG	p.Phe709Thrfs^a^17						
9	F	c.294dupA c.1825C > T	p.Leu99Thrfs^a^92	Sporadic	3 years and 8 mo	Seizures, microcephaly, difficulty swallowing, visual impairment, nystagmus, ataxia, and frequent episodes of autonomic dysregulation axial hypotonia, appendicular hypertonia, global developmental delay, motor development delay	Normal	3.5 years: progressive cerebellar and brainstem atrophy	Hanes et al. (2015)
			p.Arg609Trp						
10	F	c.294dupA c.803G > A	p.Leu99Thrfs^a^92	Sporadic	20 mo of age	Febrile seizures, hypertonia, nystagmus, esotropia, arrested head growth P10, motor development delay, developmentally delayed	Not offered	Not offered	Oatts et al. (2017)
			p.Arg268His						
11	M	c.171delG	p.Glu57Aspfs^a^7	Sporadic	15 mo old	Seizures, hypertonia, microcephaly, axial hypotonia and symmetric hypertonia, intermittent asymptomatic bradycardia and hypothermia, nonepileptic apnea, chronic lungdiseas, dry skin	Episodes of focal electrographic status epilepticus	One d of life: normal structures but subtle nonspecific foci of the cerebral white matter	Srivastava et al. (2016)
		c.419T > C	p.Leu140Pro					4.5 mo: mild global cerebral volume loss with prominence of the sulci and secondary enlargement of the lateral ventricle, normal cerebellar structures	
12	M	c.638_639insA	p.Val214Glyfs^a^189	Sporadic	Died at the age of 5 years and 9 mo due to respiratory insufficiency	Early onset epileptic encephalopathy postnatal, microcephaly, apnea, feeding problems, bradycardia, global developmental delay, maldescensus testis, left-sided club foot, and left-sided pes adductus	Focal continuous spike discharges in the right more than in the left occipital region	Thin corpus callosum, dilated internal and external cerebrospinal fluid spaces, and delayed myelination	Horn et al. (2016)
		c.1134+1G > A	Splice site						
13	F	c.1498+1G > A	Splice site	Sporadic	Died at 4 years 3 mo	Microcephaly, hypertonia, focal, multifocal motor seizures with clonic features, apnea, eye deviation to either side, clustering on awakening and drowsing, epileptic spasms, and tonic seizures	Multifocal epileptiform dischargesIctal: migrating focal seizures; seizures arising from right central region, vertex, left central, left occipital, right temporal, and left temporal region; epileptic spasms and periodic spasms, hypsarrhythmia	One m 12 d: very small hemosiderin deposition within lateral ventricles and subarachnoid spaces from previous IVH7.5 m: prominent ventricles and extra-axial CSF spaces with associated white matter volume loss, nonspecific abnormal white matter signal	Scheffer et al. (2020)
		c.1498+1G > A							
14	F	c.638dupA	p.Val214Glyfs^a^189	Sibling^a^ with NO. 25	Died at the age of 17 mo because of respiratory failure	Epileptic seizures (eye blinking and myoclonus left hand) and hypertonia, microcephaly	Continuous abnormal background pattern and multifocal seizure activity	Two mo: normal	Wolf et al. (2015)
		c.638dupA						12 mo: severe generalized atrophy, hardly any myelination	
15	M	c.1857G > A	p.Trp619^a^	Sibling with NO.8	Died at 15 mo of age	Drug-resistan seizures, microcephaly, and developmental delay	Multifocal epileptiform activity	13 mo: global cerebral and cerebellar atrophy	Smith et al. (2016)
		c.2125_2128delTTTG	p.Phe709Thrfs^a^17						
16	F	c.1359_1361delCCT	p.Leu454del	Sporadic	Died at 14 mo	Microcephaly, hypertonia, focal motor clonic seizures, migrating between hemispheres	Multifocal epileptiform discharges, discontinuous backgroundIctal: migrating focal seizures from one region to another, most frequent onset from the right posterior quadrant, other onsets in the left posterior region and left frontocentral region	Three d: small right occipital subdural hemorrhage18 d: hemorrhage resolved	Scheffer et al. (2020)
		c.1395G > C	p.Thr465Thr						
17	M	c.1313_1314delAG	p.Gln438fs	Sibling^a^	Died at the age of 12 mo	Polymorphic seizures and hypertonia, microcephaly	Generalized and focal sharp and spike waves	The myelination pattern was appropriate for the patient’s age, subarachnoid space was slightly widened	Szymańska et al. (2018)
		c.1313_1314delAG							
18	F	c.964C > T	p.Gln322^a^	Sibling with NO.27	Died at 10 mo	Microcephaly, hypertonia, focal clonic seizures with apnea, tachycardia	Multifocal epileptiform discharges, discontinuous background intermittentlyIctal: migrating focal seizures; central, right occipital spread to the left occipital region, left temporal spread to the left hemisphere then the right hemisphere, bi-occipital onset	Two d: mild thinning of the corpus callosum 2 m 10 d: mild thinning of the corpus callosum, increasing prominence of CSF spaces, likely ex vacuo dilatation	Scheffer et al. (2020)
		c.2284C > T	p.Gln762^a^						
19	M	c.2230_2237dupAACACTGC	p.S747Tfs^a^36	Sporadic	Died at the age of 10 mo	Drug-resistant seizures, hypertonia, microcephaly	4–6 Hz theta background activity, bilateral frontotemporal sharp waves and 8–10 Hz alpha waves during clinical seizures	Initial: normal, 3 mo: cerebral and cerebellar atrophy and thinning of the corpus callosum	Celik et al. (2017)
		c.2230_2237dupAACACTGC							
20	M	c.1499-1G > T	p.Glu500Alafs^a^36	Sporadic	Died at the age of 7.5 mo	Seizures (myoclonic, tonic and clonic migrating focal), hypotonia, micrognathia, microcephaly, down-slanted palpebral fissures, myoclonic jerks, apnea, and bradycardia	Generalized epileptiform activity, migrating focal epileptiform activity, and background deceleration	Atrophic corpus callosum, hypomyelinisation, brainstem, and cerebellar vermis hypoplasia	Colak et al. (2020)
		c.1499-1G > T							
21	M	c.233G > C	p.Arg78Pro	sporadic	died at the age of 7 mo due to respiratory infection and malnutrition	myoclonic seizures, paroxysmal convulsions, hypertonia, hyperactive deep tendon reflexes, small and asymmetrical frontal bones, overlapping cranial sutures, recurrent respiratory tract infections, dysphagia	Initial: more sharp wave discharges in the left forehead-parietal region than in the right forehead-parietal region 2mo: focal sharp wave discharges and spike and slow-wave complexes in the left forehead-temporal region	brain magnetic resonance imaging indicated that the bilateral frontal and temporal subarachnoid space was widened, and the corpus callosum was thin	Li et al. (2021)
		c.233G > C							
22	M	c.1173delG	p.Leu391fs	Sibling with NO.23	Died at the age of 6 mo due to cardiac arrest	Myoclonic seizures, hypertonia and contractures, arrested head growth, inability to swallow, and bouts of apnea-bradycardia, cardiac arrest	Bilateral epileptic activity with bilateral discharges	Normal	Straussberg et al. (2015)
		c.1173delG							
23	F	c.1173delG	p.Leu391fs	Sibling with NO.22	Died at the age of 5 mo due to cardiac arrest	Myoclonic seizures, hypertonia and contractures, arrested head growth, inability to swallow, and bouts of apnea-bradycardia, cardiac arrest	Sharp waves and bilateral spikes predominantly over the right hemisphere	Normal	Straussberg et al. (2015)
		c.1173delG							
24	F	c.1395G > C	p.Thr465Thr	Sporadic	Died at 10 weeks of age	Progressive encephalopathy with refractory seizures, hypertonia, episodic apnea, microcephaly, dysmorphic features	Diffuse encephalopathy, with frequent ictal activity from multiple cortical areas	Mild thinning of the corpus callous and delayed myelination	Van Ommeren et al. (2018)
		c.1395G > C							
25	M	c.638dupA	p.Val214Glyfs^a^189	Sibling^a^	Died at the age of 2 mo due to severe necrotizing enterocolitis grade III	Epileptic seizures (loss of consciousness, tonic posturing, myoclonus), hypertonia, microcephaly, mild hypotonia	Burst-suppression pattern, with long suppressions (10–15 s), multifocal negative sharp wavers	Not offered	Wolf et al. (2015)
		c.638dupA		with NO.14					
26	M	c.1120G > T	p.Glu374^a^	Sporadic	Died at 2 mo	Microcephaly, hypertonia, myoclonic seizures, focal clonic seizures migrating between hemispheres, excessive startle from day 1	Multifocal epileptiform discharges, discontinuous backgroundIctal: myoclonic seizures, clonic seizures, facial clonic movements, with migration from the right posterior occipital region to the left posterior region	17 d: small subacute subdural hemorrhage along the tentorium with left parietal bone cephalhematoma	Scheffer et al. (2020)
		c.1120G > T							
27	F	c.964C > T	p.Gln322^a^	Sibling with NO.18	Died at 34 d	Focal motor seizures, microcephaly, hypertonia	Multifocal epileptiform discharges, discontinuous backgroundIctal: focal seizure migrating from the left central region to the right hemisphere	3 d: asymmetric T2 signal in deep posterior parietal white matter bilaterally, small subdural hemorrhages in the posterior parietal region and posterior fossa, focal area of subarachnoid/pial hemorrhage in the posterior fossa adjacent to the tentorium on the right side 3 w: poor opercularization of Sylvian fissure in the frontotemporal region, hemorrhages unchanged	Scheffer et al. (2020)
		c.2284C > T	p.Gln762^a^						
28	M	c.2041G > T	p. E681X	Sporadic	Died at 6 d old	Intractable focal seizures, microcephaly, rigidity, apnea, and congenital heart disease	Not offered	Not offered	Pourahmadiyan et al. (2021)
		c.2041G > T							

EEG = electroencephalogram.

MRI = serial magnetic resonance imaging of the brain.

d = day.

mo = month.

a In “Pedigree” = sibling with similar symptoms has died without exome sequencing.

Divergence is much huger for the clinical–genetic correlation. It has been mentioned that the phenotypic spectrum of BRAT1-associated disorders is associated with the domain, localization, type, and zygosity of the identified variant (13) (Dudding et al., 2004). According to Table 1, the variant type rather than the variant domain is closely related to the phenotype severity. For example, homozygous variants R78P and p. V62E, both located on the apoptosis-related N-terminal CIDE (cell death-inducing DFF-45-like effector) (Lugovskoy et al., 1999; Choi et al., 2017), caused typical RMFSL (Li et al., 2021) and mild NPCA (Lugovskoy et al., 1999), respectively. Complex mechanisms such as the mutation affecting one or more as unidentified activities of this protein (Kurosaki and Maquat, 2016) or associated with BRAT1-related pathways might be involved. It may modulate the severity rather than simply disrupt the mitochondrial function and the ATM kinase activity. As to the variant type, individuals with BRAT1 biallelic null variants usually lead to severe symptoms and are mostly fatal at the early stage. However, in biallelic BRAT1 missense variants, phenotypic variability is huge, ranging from RMFSL to NPCA. Interestingly, we noticed that in individuals with biallelic BRAT1 gene null variants, females always exhibited a milder phenotype than males (Mundy et al., 2016; Srivastava et al., 2016), even for siblings who carried the same variants. The special phenotypic divergence cannot be explained simply by variable penetrance or genetic backdrop heterogeneity. It is hypothesized that other sub-equivalent genes located on the X chromosome have some effects of the “female protective model” in neurodevelopmental disorders (Jacquemont et al., 2014). The mechanism is yet to be further confirmed.

The variant p. Leu236Cysfs*5 resulted truncation after the first 236 residues, and half of the protein was most likely to lose the function. The trans variant c.1014A > C translates to p. Pro338Pro, which has not been functionally silent. It generated an aberrant transcript with intron 7 retention by affecting the splicing accuracy. Intron retaining can reduce gene expression at the post-transcriptional level and thereby impose an additional level of gene regulation, such as the degradation of mRNA transcripts *via* nonsense-mediated decay (NMD) and the regulation of nuclear mRNA export (Low et al., 2015; Schmitz et al., 2017). Wild-type BRAT1 was diffusely expressed in the cytoplasm and the nucleus (Li et al., 2021). As-retained intron transcripts accumulate in the nucleus (Boutz et al., 2015), which might also reduce the amount of cytoplasmic BRAT1 available for the downstream events.

The poor efficiency of mRNA transcription, rather than completely abolished BRAT1 protein in a truncating genetic backdrop, made the BRAT1 protein synthesis in the early life insufficient to maintain the good development of the cerebellum, which may underlie the etiology of the mild, nonprogress phenotypic form of *BRAT1*-related neurodevelopmental disorders. c.1014A > C is the first pathogenic synonymous variant identified in the BRAT1 gene, which is associated with autosomal recessive NPCA. Before this, there was a synonymous substitution at c.1395G > C (p.Thr465 = ), which had been reported in the severe phenotypic form of RMFSL (24). Although the mechanism has not been clarified, it conjectured that there exist multiple exon skipping, mRNA degradation, and complete deletion of BRAT1 protein. Novel synonymous variants in *BRAT1* should never be ignored as silent sound in diagnosis, which occasionally shed light on the underlying pathogenesis of the disease.

## Conclusion

Our results not only broaden the mutation/phenotype spectrum of *BRAT1* but also contribute to comprehend possible pathogenic mechanisms of *BRAT1*. It is beneficial to specific genetic counseling and timely perinatal management.

## Data Availability

The datasets for this article are not publicly available due to concerns regarding participant/patient anonymity. Requests to access the datasets should be directed to the corresponding author.
